# A qualitative geographical information systems approach to explore how older people over 70 years interact with and define their neighbourhood environment

**DOI:** 10.1016/j.healthplace.2015.10.002

**Published:** 2015-11

**Authors:** Sarah Milton, Triantafyllos Pliakas, Sophie Hawkesworth, Kiran Nanchahal, Chris Grundy, Antoinette Amuzu, Juan-Pablo Casas, Karen Lock

**Affiliations:** a Department of Health Services Research and Policy, Faculty of Public Health and Policy, London School of Hygiene & Tropical Medicine, London, UK; b Department of Social & Environmental Health Research, Faculty of Public Health and Policy London School of Hygiene & Tropical Medicine, London, UK; c Department of Non Communicable Disease Epidemiology, Faculty of Epidemiology and Community Health, and British Womens Heart and Health Study, London School of Hygiene & Tropical Medicine, London, UK

**Keywords:** Neighbourhood, Ageing, Qualitative geographical information systems, Methods, Space, Environment

## Abstract

A growing body of literature explores the relationship between the built environment and health, and the methodological challenges of understanding these complex interactions across the lifecourse. The impact of the neighbourhood environment on health and behaviour amongst older adults has received less attention, despite this age group being potentially more vulnerable to barriers in their surrounding social and physical environment. A qualitative geographical information systems (QGIS) approach was taken to facilitate the understanding of how older people over 70 in 5 UK towns interact with their local neighbourhood. The concept of neighbourhood changed seasonally and over the lifecourse, and was associated with social factors such as friends, family, or community activities, rather than places. Spaces stretched further than the local, which is problematic for older people who rely on variable public transport provision. QGIS techniques prompted rich discussions on interactions with and the meanings of ‘place’ in older people.

## Highlights

• Neighbourhoods become increasingly important to older people's wellbeing.
• Older people associate neighbourhood with people and activities rather than places.
• Neighbourhoods are dynamic; change seasonally and over the lifecourse.
• Qualitative GIS produces rich data on older people's interactions with place and space.

## 1 Introduction

There has been growing interest in research exploring the relationship between the built environment and different aspects of individuals’ health, with studies showing the impact of both the physical and social environment, and the methodological challenges of understanding these complex interactions (Rydin et al., 2012, Kestens et al., 2012, Booth et al., 2005, Macintyre et al., 2002, Giles-Corti and Donovan, 2002). Within this field, the impact of the neighbourhood environment on health behaviours and outcomes of older adults has received relatively little attention, despite potentially being more vulnerable to barriers in their surrounding social and physical environment (Clarke and Nieuwenhuijsen, 2009). As people age, and their mobility and cognition declines, their residential neighbourhood environments may become more relevant to their health and wellbeing (Yen et al., 2009). Most research on ageing in place focuses on the home or retirement communities. Little research has looked at public spaces of ageing to contextualise the lives and wellbeing of older adults (Gardner, 2011).

### 1.1 Conceptualising ‘neighbourhoods’

Within health and place research, definitions of an individual's local environment, and concepts of neighbourhood vary widely between studies. To date, much of the research within this field has relied on defining an individual's local environment using fixed spatial units, often administrative boundaries, reflecting the ease of data collection at this level (Perchoux et al., 2013). Within the environmental health literature, geographic information systems (GIS) are used to define areas of influence based on a local area centred on an individual (an ‘ego-centred’ neighbourhood), with boundaries defined by a given distance threshold (Chaix et al., 2009). Buffers may be circular or elliptic zones, or road networks, and the size of the buffer will be highly population- and hypothesis-dependent (Perchoux et al., 2013). Often studies apply an operational definition, such as the distance walked in 10 min.

It is increasingly recognised that these definitions may underestimate any association with the local environment as they do not accurately represent each individual's unique spatial experience (Perchoux et al., 2013, Cummins et al., 2007, Kwan, 2012). Indeed the conceptualisation of ‘place’ and ‘neighbourhood’ has been highlighted as one of the weakest theoretical areas in the field of health and environment research (Matthews, 2008). Residential neighbourhoods, operationalized as static administrative areas such as census tracts, postal code areas or buffer areas around individuals’ home addresses, might not accurately represent the actual areas that exert contextual influences upon health; for example opportunities for physical activity and the diversity of food available near to work or social activities could be more important influences on health than factors nearer to home environments (Kwan, 2012). However, social contexts such as friends, families and peers are not in themselves geographically defined, and cannot be easily delineated as geographic areas with precise boundaries (Macintyre et al., 2002).

### 1.2 The use of QGIS methods to understand space and place

Various qualitative approaches have been used to explore how people perceive their neighbourhood or spaces they commonly use in their local area. These include tools such as photovoice and photo elicitation interviews (Plane and Klodawsky, 2013) or participant diaries and interviews, (Latham, 2003) all of which aim to facilitate interview discussions and reach a deeper understanding of the interactions between people and place. Early linking of GIS and qualitative research was made by participatory GIS researchers, often in planning, and has now been developed with a variety of different qualitative methods used (Kwan and Ding, 2008). GIS has been used to triangulate qualitative methods (Pavlovskaya, 2006, Pavlovskaya, 2002), or in order to produce discrepancies in different types of data, that serve to raise research questions that otherwise would not be apparent (Kwan and Ding, 2008). ‘Geo ethnography’, developed by Matthews et al., combines ethnography with geospatial and GIS methods (Matthews et al., 2005). A US multi-site study on welfare, families and children, found GIS generated mapping was insufficient, and ethnographic data was needed to complement and understand details about the cultural meanings of place and the political and sociocultural influences on the daily lives of families. Conversely the study also found that the maps produced by GIS appeared more powerful than narrative ethnographic accounts for research translation practices, including communicating with policy makers (Matthews et al., 2005).

For Kwan & Ding, GIS techniques can also be combined with the analysis and interpretation of qualitative narrative materials such as oral histories, life histories and biographies – in what they call ‘geo narratives’ – in order to account for the full spectrum of contextual influences on behaviours or health outcomes (Kwan and Ding, 2008). In a US study of racialised and gendered urban space in post 9/11 US, activity diaries and oral histories were complemented with maps of their study area that participants drew upon to indicate locations that were part of their daily lives and where they felt safe or unsafe, pre and post 9/11 (Kwan, 2008). Knigge & Cope, building on feminist perspectives of GIS, combine spatial data visualisation with qualitative grounded theory at the analysis stage of research, in what they call ‘grounded visualisation’ (Knigge and Cope, 2006).

This paper presents the results of a pilot study that explored the applicability of using QGIS techniques to facilitate the understanding of how older people in the UK understand, and interact with, their local neighbourhood; and how these public spaces of ageing interacted with their perceptions of health and wellbeing. The pilot study was part of a larger cross-sectional study of the association between the neighbourhood environment and health behaviour in older people, a second objective of the pilot being to inform the analysis of the national study by understanding the different scales upon which various environmental features operated for older adults.

## 2 Materials and methods

This pilot study included a sample of 14 women and men over the age of 70 who lived in 5 English towns. Participants were initially drawn from the British Women's Heart and Health Study (BWHHS), a prospective cohort study of women aged between 60 and 79 years randomly selected between April 1999 and March 2001 from general practitioner lists in 23 British towns. Full details of the BWHHS have been previously reported (Lawlor et al., 2003). For this pilot, participants were selected from a sub-sample of 170 women in wave 3 (2007) who had valid accelerometry data measuring physical activity for 7 days. The five towns were selected to represent a socio-economic range, by ranking all BWHHS towns high to low by mean Index of Multiple Deprivation score (IMD: https://www.gov.uk/government/collections/english-indices-of-deprivation). This resulted in the selection of Guildford, Newcastle-under-lyme, Bedford, Wigan and Bristol. A random sample of 29 women were identified and invited to participate in the study. The sampling was based on their location of residence (80% centrally located in the town, 20% at the town periphery based on the Office of National Statistics –ONS- urban/rural classification), their physical functioning (80% “third age”, 20% “fourth age” (Laslett, 1996)) and the index of multiple deprivation (IMD) score for the lower layer super output area (LSOA) where participants were residing (50% most deprived/50% least deprived based on the 2000 IMD). Ten women responded and were approached for informed consent. Using a snowball sampling technique, women enroled into the pilot study were then asked to provide information on male contemporaries who they did not live with, and these were then approached for informed consent. In addition, due to difficulty in recruiting men into the study, male recruitment was attempted via community groups for older people in the same study towns. Ethical approval for the study was granted by the London School of Hygiene Ethics Committee and the NHS National Research Ethics Committee (7213-01) and NHS LREC (MREC/02/2/91).

**Fig. 1:**
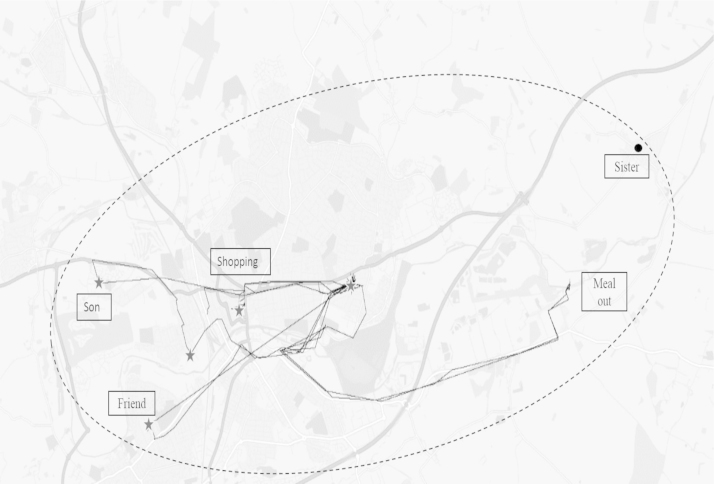
Example of an overview activity space map generated from 7 day GPS data. Figure shows an example of an ‘overview’ activity space map that was used for interviews. Maps were generated from GPS data in ArcGIS and exported to Google Maps. Solid lines represent GPS tracts recorded during the 7 day period and stars represent stops of more than 10 min at a single location. The dashed line represents the interviewee's view of their ‘neighbourhood’, which was annotated onto the maps during the interview and stretched further than the GPS tracts.

Participants were visited by a member of the study team at the start of the study period and shown how to use a small GPS device [102-Nano Covert Magnetic Tracker]. Participants were asked to wear the GPS device for a 7 day period (all day except for when they are asleep) in order to record the places that were visited during this time. Locations were recorded by the GPS device in 90 s intervals. The location data were then mapped with GIS software (ArcGIS 10.3) and exported to Google Maps. The aim was to provide a visual presentation of participants’ daily routine place interactions. Between 10 to 20 maps were produced for each participant. These included higher resolution maps looking at specific events, visits and routes on a day, daily maps and overall activity maps reflecting all data captured across the 7 day period. Stops were defined as locations where individuals were for more than 10 min and these were discussed at interview (Fig. 1). Additionally, participants were asked to keep a brief diary of their movements and main activities over the study period.

After the 7 day GPS-wearing period, the devices were mailed back to the research centre by participants in pre-paid envelopes. Within 2 weeks of wearing the GPS device, a follow up in-depth interview was conducted with each participant in their own home using the daily and summary activity maps and diaries for each individual. Data were collected and interviews took place between July and October 2014.

The in-depth interviews initially discussed how individuals found the practice of wearing the GPS device and then focused on a more detailed discussion of the participant's activity and neighbourhood, prompted by the activity space maps. Participants were prompted to discuss how well the maps represented their view of their’neighbourhood’ and were encouraged to write on the maps during the interview to annotate them in order to describe activities and spaces they often engaged with in their neighbourhood. Interviews were transcribed verbatim and transcripts were analysed using thematic content analysis (Green and Thorogood, 2013), with a mixture of inductive and deductive coding, to identify emerging themes. Deductive themes were broad, overarching and relevant to the study questions, for example ‘definitions of neighbourhood’. Within these themes however analytical themes such as ‘the loss of the local’ and ‘dynamic neighbourhoods’ were developed, again with inductive subthemes within each, for example for the latter, ‘seasonal neighbourhoods’ and ‘changes over time’. NVivo10 software was used to organise the transcripts and aid the analysis. At different stages of the research, a total of three members of the team read the transcripts and discussed the coding scheme and emerging themes.

## 3 Results

A total of 10 women and 4 men participated in the pilot study. Participants ranged in age from 75 to 88 years, all self-identifying as White British. When compared to our selection criteria, women aged over 80 years living in less deprived areas were over-represented. However, our study participants came from a range of socio-economic backgrounds and physical environments (urban and semi-rural) across all 5 study towns (Table 1). A surprising finding was that all of the study participants had lived in the same locality, if not the same house, for a substantial amount of time – ranging from about twenty years to the individual's entire life. Even if they had moved house, they had moved very close by within the area. The majority of the participants had family, usually sons and daughters and sometimes siblings, living very close by.

The majority of participants in the study found using the GPS devices to be practically unproblematic as they wore the small devices around their necks, or kept it in their handbags or pockets. There was also little to no concern from participants regarding the perceived potential implications of wearing a GPS tracker, i.e. their activities being recorded over the course of a week.

The participants were able to deal with practical aspects of using the GPS devices including recharging them during the week, and there were only a small number of technical issues. One of the GPS devices stopped collecting data and had to be replaced, and for 7 participants, missing data was found for some parts of the 7 days when activity maps were generated. Missing data were due to participants forgetting to wear and use the device or device problems (eg. loss of signal, battery problems). Data for at least 5 days were collected from all participants. Even if some participants had missing data for some of the 7 days they were still used as the purpose of the maps was an interview tool, not as a complete representation of an individual's activity patterns over a week.

During the interview, an individual's view of their own ‘neighbourhood’ was drawn onto the activity space maps and the discussion of the maps was a useful way of triangulating information on neighbourhood and activity between the participants’ own perceptions and views, the data from the activity diary and the 7 days recorded by GPS. Framing the interviews in this way gave a deeper sense of daily life and routines – for example, annotating the maps often jogged the participants’ memories and they recalled weekly visits or routines that would have otherwise been lost if solely using an interview. Maps complemented the interview but could also be used as standalone data; the overview activity space maps aggregated from the 7 days of GPS data were the most useful as they provided a sense of the overall ‘neighbourhood’ for a wide range of activities over the course of one week. However, as the boundaries were often being redrawn by participants during interviews, the subjective definitions of neighbourhood space could be more fully accounted for combining interview data than by using the maps alone.

### 3.1 Subjective neighbourhoods: defining boundaries through people and activity

When facilitating the interviewees to discuss subjective understandings of what they thought of as their neighbourhoods, the conversation was flowing and rich. ‘Neighbourhood’, rather than being defined by place, was defined by people and regular activities in their lives.

“*I feel like, neighbourhood is made up of people rather than erm an area really*”. (Woman, 80+, semi-rural).

Discussions of the subjective definitions of neighbourhoods – how people interacted with places and what this meant to them-were facilitated by the use of the individual activity space maps (particularly the 7 day overview map). Whilst trying to identify the boundaries of an individual's neighbourhood, although an interview would commence by using the outer limits of the maps, all participants actively redrew the boundaries of the space that they considered as their neighbourhood.

In this study, most of the participants in all study towns perceived their neighbourhood to stretch further than what was represented on their individually activity space map i.e. further than the places that they had visited during the course of the week that they were monitored. Often the boundaries of subjective neighbourhoods were expanded out towards where their families, or other important social contacts lived. For example, in one case the participant redrew the boundary of her neighbourhood to explicitly include where her sister lived (Fig. 1).

Aside from defining neighbourhood through close social contacts, participants in all study towns mainly defined their neighbourhood by the regular activities that formed integral parts of the participants' lives. For example, dog walking was a key component of several participants' lives and this framed how they described their neighbourhood, and community. For these participants dog walking was part of a regular, daily routine, which helped them exercise, but additionally, and for some more importantly, helped them access a friendly local community. A key part of neighbourhood for these people was defined by where they could go walking with their dogs, but also linked with the people they met regularly through these activities;

*“It's marvellous for meeting people… and people in houses you know, they notice me going by and they’ll wave because they know it's my time, it's quite sweet. So I have made quite a few friends just through the dog … you see we used to do it with our children at school. The children grow up and go away and you’ve got no way of doing it, but with a dog you do.. that's sort of the eight o’clock meeting”* (Woman, 70–79, semi-rural).

In discussions of neighbourhood, many participants’ highlighted the importance of community associations or institutions they frequented regularly. These included community groups, walking groups, University of the 3rd Age groups, churches, golf clubs (the latter especially for some of the male participants). Participants stressed that the importance of these was more about the people than the content of what they were actually doing;

*“I know it sounds a bit funny, but we’re not really religious people, it's more like a club. It's a jolly good church this… they’re nice people, so we, we see quite a lot of them”* (Woman, 70–79, semi-rural).

*“it's not just playing golf, it's the… social emphasis that golf has with meeting a group of your pals… it's not just golf… I wouldn’t be without it for the world, I’d be lost I think”* (Man, 70–79, semi-rural)

Another key way of participants defining (and during interviews re-defining) the boundaries of their neighbourhood was by local places of interest such as parks, historic houses or garden centres, which they would visit regularly for walks, or as a pleasant place to have lunch or drinks;

*“I would say the garden centres because they give an informal place where one can get refreshments reasonably. They’re not too expensive to go to and pleasant surroundings”* (Woman, 80+, semi-rural).

Defining neighbourhoods through people and regular activities also meant that participants’ perceptions of neighbourhoods were dynamic and changed over time. Interestingly, a few participants commented on the fact that the concept of their neighbourhood was seasonal. For one man, his concept of his neighbourhood reduced in size in the winter, when it became too cold for him to play golf and spend time outdoors. For one woman, her neighbourhood got much smaller in the summer months, especially August, when most of her regular activities which were organised by the University of the Third Age stopped for the summer holiday period. Participants also clearly described how their perception of neighbourhood had changed over time. One participant described how her neighbourhood, and community, had been defined in the past by schools – both her own as the quote below describes, and then that of her children;

*…you see in my day you went out of your village to school and then … that becomes part of your neighbourhood* (Woman, 70–79, semi-rural).

Now she was older and as schools were no longer part of her life, the boundaries of her neighbourhood had been conceptually reshaped so as not to include them.

Although participants had a strong sense of what they perceived as neighbourhood, there was difficulty within the interviews when trying to prompt the participants to think through ‘objective spaces’, for example spaces defined in terms of specific distances from their homes, even when the prompts were quite direct. Unless the immediate area around their house was being discussed it was often difficult to clarify answers about, for example, how they interacted with places within one mile compared to further distances away. This appeared to be because study participants did not conceptualise everyday spaces in this way – it simply did not match or align with their everyday experience of, and natural ways of defining and describing, the spaces around them.

### 3.2 The importance of local facilities to concepts of neighbourhood

Most of the participants described having to travel outside of their immediate local area for most of their activities or facilities – it was common to hear participants describe their immediate neighbourhood environment as lacking in both essential and useful facilities for all ages, these commonly included shops, pubs and playgrounds; “*there*'s *nothing in this area… not even a swing for a child… nothing for old or young*” (Woman, 80+, urban);

Interviewer: *“And do you spend much time, like, close to your house?”*

Participant: *“No, there's nowhere to go. I’m round my house, do my gardening and that's about all”* (Man, 80+, urban)

For most of the participants shopping for food was discussed as one of their main weekly activities. The interview discussions not only included the current places where they shopped but naturally covered the changes in the places and ways people shopped over time. This in turn appeared to have shaped perceptions of how shopping was related (or not) to their ideas about their neighbourhoods. It was rare in this study for people to use their local shops, despite describing having done so in the past. They described how this signified a change in their relationships with neighbourhood and neighbours. For many participants, local facilities, such as local shops, no longer existed in their immediate vicinity and whereas shopping used to form an important part of the participants’ concept of neighbourhood, the changes in their local environments meant that shopping was now not necessarily linked to their definitions of neighbourhood. For most participants, their current shopping practices entailed using either out of town, large supermarkets or doing smaller shops, on the way to and from wherever they happened to be going to. Thus although shopping is still a main activity it is often added onto other activities, for example, people would shop for food whilst on the way home from somewhere else rather than being the main focus of a journey; “*I, now I tend to shop where I am, if you understand what I mean”* (Woman, 70-79, urban).

*“There's a bakers shop closed down, that's the last bakers shop… the last of the family baker… it's all out of town shopping now”* (Man, 80+, urban)

This seemed to be part of a wider trend, with participants also lamenting the loss of local post offices and pubs which they had previously used. In some areas they had been completely lost, while in other places they had been replaced by bigger, less personal venues or other services that did not appeal to the older people. Whereas participants perceived that local shops, pubs and post offices had in the past fostered neighbourhood relationships, a sense of community identity had been lost with the loss of these facilities;

*“I’ll tell you, years ago, before McDonalds was built here, was a massive pub. And it, it was virtually the hub of the community. But that closed … And since then, the community spirit seems to have dropped around here. People just say hello, and that's it, are you doing your gardening again and, you know. I see people going along to the paper shop every day and I’ve seen them for years and I don’t know who they are.”* (Man, 80+, urban)

*“It's terrible, and there's such a lot of old people that are lonely. Really very, very lonely. Especially now because, you see, everybody's working, there's nobody about… when the Post Office was on [nearby] road we used to get a lot of people walking down. Then they moved it into the, well it's a One Stop now, our convenience store, and um of course people go the other way now.”* (Woman, 70–79, semi-rural).

It was recognised that having local facilities was now unusual.

### 3.3 The importance of transport to neighbourhood access

Due to the perceived lack of immediate local services and resources, participants highlighted the increasing importance to them of transport in being able to access their wider neighbourhood. It was common within most interviews to discuss the status of public transport in their area. There was a range of views, either public transport was perceived as an important service that facilitated their ability to get to the nearest shops, healthcare or other services, or as something that was lacking and thus hindered their ability to undertake activities. Where public transport was discussed as being lacking, participants described relying heavily upon their cars or those of others. A common concern was that there would come a time in their lives, for many in the near future, when they might not be able to drive regularly, or at all, due to their age and health conditions, which would limit the places they could access. Having family living close by made this transition away from driving easier for some, but lack of public transportation remained an important barrier to neighbourhood access discussed by most participants, particularly those in the more semi-rural areas. This left some feeling isolated at home;

*“I think one values being able to use a car tremendously… I know people of my age who have had to give up driving, and they do find that just alters their life completely… I think it must be very hard to accept… you’re giving up driving at a time when you most need the car because a lot of people don’t find it so easy to get around at that stage”* (Woman, 80+, semi-rural).

*“Public transport around here's awful. I mean a friend of mine lost his license for six months, nearly drove him mad… it's useless. Um so other than having his wife drive him around he was housebound… a car is absolutely vital”* (Man, 70–79, semi-rural)

One man described his concerns about reduced mobility and ability to access the wider facilities needed once he had to give up driving. He enjoyed his local, semi-rural neighbourhood where he lived with his wife, but was concerned about becoming *’far too isolated’* when no longer able to drive. His wife had been diagnosed with Alzheimers and had herself stopped taking the bus alone due to feelings of vulnerability. He described his plan during the interview of moving into a house in a nearby town when he could no longer drive,

*“ to get isolated in this house would be really hopeless… it's much more convenient [in town], near shops, banks… far more buses.*” (Man, 80+, semi-rural)

Where bus routes operated regularly, participants were enthusiastic about using the buses. One described how there was even a sense of a neighbourhood on the bus;

*“Everybody was saying, oh gosh we don’t often see you on the bus! Because it's quite a social occasion for a lot of these ladies… because they all meet up, you know, if they’re on their own and they don’t see many people, it's quite nice.*” (Woman, 70–79, urban)

For some people there had been closures of local bus routes, leading to expressions of concern about their future ability to continue doing regular activities they valued. One couple described how they used to catch the bus regularly to go for a short walk, but that they could no longer do this because the bus route had been closed down;

*“We used to catch a bus and go up on the bus and have a little walk, have an ice cream, have a rest, and come back. But you can’t do that now because we’ve got no buses.”* (Woman, 80+, urban).

## 4 Discussion

There is little research that has explored the influence of public spaces, compared with residential places, to understand the daily practices and wellbeing of older adults (Gardner, 2011). However, neighbourhoods are extremely important places of ageing, where going outside to interact with the material and social neighbourhood is essential to wellbeing and self-identity among older adults (Peace et al., 2005a, Peace et al., 2005b). There are insufficient empirical studies within this field that relate specifically to the barriers and facilitators of neighbourhood environments encountered by older people (Yen et al., 2009).

The rich discussion prompted by the interviews in this study highlighted some important aspects about the concept of ‘neighbourhood’ in this age group, which can in turn inform the design and analysis of larger epidemiological studies. In general, neighbourhoods were viewed as being defined by people and activities rather than geographical space and were often larger than the areas captured by the activity space maps; with participants actively redrawing the boundaries of what they perceived to be their neighbourhoods during the interviews. Previous research has identified family and non-family relationships as one of the most important areas of support for older people (Walker and Hiller, 2007, Nocon and Pearson, 2000). Other studies have also recognised the importance of the ‘social space’ rather than just physical characteristics (Wiles et al., 2009) and neighbourhood has been called a “process in which social relations and identities are constructed” (Gardner, 2011).

This study also highlights the importance of considering the dynamic nature of individuals’ relationships with their local area, with the definition of, size and composition of neighbourhoods perceived as changing with the seasons and over the lifecourse. Many participants described their neighbourhood becoming more restricted as they get older and less mobile. Shopping habits were described as having changed over time with much more convenience shopping conducted wherever the individuals were on a given day, replacing both local shopping and the weekly supermarket shop, and some activities were described as being seasonal. These important daily, annual and longer term changes are often not considered in eco-epidemiologic research. Transport was highlighted as a vital facilitator to accessing neighbourhood activities and to being a fully integrated member of the community, with many individuals concerned that ageing would lead to a significant loss of community access if public transport provision was insufficient for their needs.

Researchers have conceptualised local environments as composed of multiple factors influencing the macro-level (legislation and policy), the physical environment level (access and availability of foods at home, work, shops etc.) and the social environment level (social and cultural norms) (Chow et al., 2009). Much of the literature in this field is focussed on quantitative associations between objectively measured aspects of the local environment and health or health behaviours (Cummins et al., 2005). Other studies have explored how individual's perceptions of their environment may be related to health (Bowling et al., 2006, Poortinga et al., 2007). The importance of considering environments encountered outside of an individual's immediate vicinity have been recognised with authors such as Cummins describing the “local trap” (Cummins, 2007) or “residential trap” (Chaix, 2009) suffered by many environmental epidemiology studies. Others have argued that this methodological concern may be less important amongst the older age group (Perchoux et al., 2013), who often have fewer responsibilities and/or have functional limitations leading to increasing amounts of time immersed in their neighbourhood (Gardner, 2011).

Individuals in the current study struggled to define their neighbourhoods or activities within geographical parameters such as a particular distance from their home. Both this finding, and the fact that neighbourhoods were generally perceived in this study as larger than their common activity space maps, may have important implications for studies that investigate individuals’ perception of their ‘local environment’ – highlighting the importance of an individual's definition of ‘local’ or neighbourhood in context. The current study suggests that even within this age group it is important to consider environmental exposures at different geographical distances, considered further than the ‘local’, as facilities such as shops were often accessed at various distances to the participants’ home. As others have highlighted, older people are a heterogeneous group and many are very active and can easily drive or get transport to distant locations (Yen et al., 2009). The loss of local facilities, and by extension local communities, was described by participants in this study as a fairly recent change, encompassing shops, post offices, pubs and libraries. The lack of accessible public transport options were seen as a barrier to health and wellbeing when considering access to shops and services if people were no longer able to drive.

The purpose of this qualitative study was also methodological, exploring the applicability of using GIS and GPS technology with people over 70 to facilitate understanding of how older people in the UK perceive their local neighbourhood. Combining spatial data with in-depth interviews allowed a much greater understanding of how and why individuals interact with their local environment. Participants were happy to wear tracking devices and both participants and researchers found the maps a very useful interview tool. Using individuals’ activity space maps alongside in-depth interviews was a particularly useful technique when trying to capture behaviours and viewpoints that were part of everyday life, and may feel so mundane to the participant that they are difficult to capture in a traditional interview only approach (Bell et al., 2015). The use of activity space maps ensured participants were reminded of common activities, which might otherwise be easily overlooked, leading to a richer discussion and analysis. Bell et al. utilised a similar approach to explore the specific environmental interaction of green-space usage (Bell et al., 2015). Encouraging participants to redraw the boundaries of their neighbourhoods onto their maps prompted an in-depth consideration of what might have otherwise been an abstract concept to consider. As with other studies, participants found it difficult to conceptualise neighbourhood space in terms of objective distances (e.g in number of miles), as this was not how their neighbourhood spaces were lived or made meaningful. Visualising and drawing on the map, whilst discussing activities during the interview, was a successful way of exploring how older people define and interact with space.

There are some important limitations to highlight both with this approach and with this particular study. Firstly, relying on GPS devices as the main data capture tool requires participant cooperation and a reliance on technology. We found no issues with cooperation with participants who consented to take part, however women over 80 and living in lesser deprived areas were overrepresented and this would have shaped the data in ways we were unable to explore. We were easily able to recruit women but found it harder to recruit men and we were unable to discover why men were discouraged from taking part. The difficulty in recruiting sufficient men prohibited any analysis of gender-specific views and we only recruited individuals from a single ethnic group. In addition, as with any technique that aims to capture data on everyday behaviours it should be acknowledged that a particular week may not be representative of an individual's normal life and/or behaviour may be altered as a consequence of being enroled in the study. We also had insufficient numbers to explore differences related to different functional abilities. Potential differences with gender, ethnicity and functional ability should be explored in a larger QGIS study.

## 5 Conclusion

Neighbourhood is a dynamic concept and means different things to different people at different times in their lives. Within this study of older people the definition of neighbourhood was particularly associated with social factors such as friends and family, or community activities, rather than places or facilities. Neighbourhoods were seasonal and changed over the lifecourse. QGIS techniques represent a useful method within this area to understand the activity spaces that individuals inhabit and to prompt rich discussions on interactions with and the meanings of ‘place’. Any consideration of health-place associations, and its application to policies and interventions, would benefit from a greater understanding of the interactions and definitions used by individuals, and QGIS techniques provides an approach to explore these themes in more depth.

## Figures and Tables

**Table 1:** Descriptive characteristics of the study sample.

**Selection criteria**	**Women (*n*=10)**	**Men (*n*=4)**	**All (*n*=14)**
**Age group**			
3rd age (70–79 yrs)	7 (70%)	2 (50%)	9 (64.3%)
4th age (80+ years)	3 (30%)	2 (50%)	5 (35.7%)
			
**Location**			
Centre	8 (80%)	3 (75%)	11 (78.6%)
Periphery	2 (20%)	1 (25%)	3 (21.4%)
			
**Deprivation**			
Low	8 (80%)	2 (50%)	10 (71.4%)
High	2 (20%)	2 (50%)	4 (28.6%)

## References

[bib1] Booth K.M., Pinkston M.M., Poston W.S. (2005). Obesity and the built environment. J. Am. Diet. Assoc..

[bib2] Bowling A. (2006). Do perceptions of neighbourhood environment influence health? Baseline findings from a British survey of aging. J. Epidemiol. Community Health.

[bib3] Bell S. (2015). Using GPS and geo-narratives: a metholdogical approach for understanding and situating everyday green space encounters. Area.

[bib4] Clarke P., Nieuwenhuijsen E.R. (2009). Environments for healthy aging: a critical review. Maturitas.

[bib5] Chaix B. (2009). Neighbourhoods in eco-epidemiologic research: delimiting personal exposure areas. A response to Riva, Gauvin, Apparicio and Brodeur. Soc. Sci. Med..

[bib6] Cummins S. (2007). Understanding and representing ‘place’ in health research: a relational approach. Soc. Sci. Med..

[bib7] Chow C.K. (2009). Environmental and societal influences acting on cardiovascular risk factors and disease at a population level: a review. Int. J. Epidemiol..

[bib8] Cummins S. (2005). Measuring neighbourhood social and material context: generation and interpretation of ecological data from routine and non-routine sources. Health Place.

[bib9] Cummins S. (2007). Commentary: investigating neighbourhood effects on health--avoiding the ‘local trap’. Int. J. Epidemiol..

[bib10] Chaix B. (2009). Geographic life environments and coronary heart disease: a literature review, theoretical contributions, methodological updates, and a research agenda. Annu. Rev. Public Health.

[bib11] Giles-Corti B., Donovan R.J. (2002). The relative influence of individual, social and physical environment determinants of physical activity. Soc. Sci. Med..

[bib12] Gardner P. (2011). Natural neighborhood networks — important social networks in the lives of older adults aging in place. J. Aging Stud..

[bib13] Green J., Thorogood N. (2013). Qualitative Methods for Health Research.

[bib14] Kestens Y. (2012). Association between activity space exposure to food establishments and individual risk of overweight. PLoS One.

[bib15] Kwan M.-P. (2012). The uncertain geographic context problem. Ann. Assoc. Am. Geogr..

[bib16] Kwan M.-P., Ding G. (2008). Geo-Narrative: extending geographic information systems for narrative analysis in qualitative and mixed-method research. Prof. Geogr..

[bib17] Kwan M.-P. (2008). From oral histories to visual narratives: re-presenting the post-September 11 experiences of the Muslim women in the USA. Soc. Cult. Geogr..

[bib18] Knigge L., Cope M. (2006). Grounded visualization: integrating the analysis of qualitative and quantitative data through grounded theory and visualization. Environ. Plan. A.

[bib19] Latham A. (2003). Research, performance, and doing human geography: some reflections on the diary-photograph, diary-interview method. Environ. Plan. A.

[bib20] Lawlor D.A. (2003). Geographical variation in cardiovascular disease, risk factors, and their control in older women: British women's heart and health study. J. Epidemiol. Community Health.

[bib21] Laslett P. (1996). A Fresh Map of Life: The Emergence of the Third Age.

[bib22] Macintyre S., Ellaway A., Cummins S. (2002). Place effects on health: how can we conceptualise, operationalise and measure them?. Soc. Sci. Med..

[bib23] Matthews S.A. (2008). The salience of neighborhood: some lessons from sociology. Am. J. Prev. Med..

[bib24] Matthews S.A., Detwiler J.E., Burton L.M. (2005). Geo-ethnography: coupling geographic information analysis techniques with ethnographic methods in urban research. Cartograph.: Int. J. Geogr. Inf. Geovis..

[bib25] Nocon A., Pearson M. (2000). The roles of friends and neighbours in providing support for older people. Ageing Soc..

[bib26] Perchoux C. (2013). Conceptualization and measurement of environmental exposure in epidemiology: accounting for activity space related to daily mobility. Health Place.

[bib27] Plane J., Klodawsky F. (2013). Neighbourhood amenities and health: examining the significance of a local park. Soc. Sci. Med..

[bib28] Pavlovskaya M. (2006). Theorizing with GIS: a tool for critical geographies?. Environ. Plan. A.

[bib29] Pavlovskaya M.E. (2002). Mapping urban change and changing GIS: other views of economic restructuring. Gend. Place Cult.: J. Fem. Geogr..

[bib30] Peace S., Kellaher L., Holland C. (2005). Environment and Identity in Later Life.

[bib31] Peace S.M., Holland C., Kellaher L., Rowles G.D., Chaudhury H. (2005). The influence of neighborhood and community on well-being and identity in later life: an english perspective. Home and Identity in Late Life: International Perspectives.

[bib32] Poortinga W., Dunstan F.D., Fone D.L. (2007). Perceptions of the neighbourhood environment and self rated health: a multilevel analysis of the Caerphilly Health and Social Needs Study. BMC Public Health.

[bib33] Rydin Y. (2012). Shaping cities for health: complexity and the planning of urban environments in the 21st century. Lancet.

[bib34] Walker R.B., Hiller J.E. (2007). Places and health: a qualitative study to explore how older women living alone perceive the social and physical dimensions of their neighbourhoods. Soc. Sci. Med..

[bib35] Wiles J.L. (2009). Older people and their social spaces: a study of well-being and attachment to place in Aotearoa New Zealand. Soc. Sci. Med..

[bib36] Yen I.H., Michael Y.L., Perdue L. (2009). Neighborhood environment in studies of health of older adults: a systematic review. Am. J. Prev. Med..

[bib37] Yen I.H., Michael Y.L., Perdue L. (2009). Neighborhood environment in studies of health of older adults: a systematic review. Am. J. Prev. Med..

